# Coordinated Activity of Ventral Tegmental Neurons Adapts to Appetitive and Aversive Learning

**DOI:** 10.1371/journal.pone.0029766

**Published:** 2012-01-06

**Authors:** Yunbok Kim, Jesse Wood, Bita Moghaddam

**Affiliations:** Department of Neuroscience, University of Pittsburgh, Pittsburgh, Pennsylvania, United States America; Université Pierre et Marie Curie, France

## Abstract

Our understanding of how value-related information is encoded in the ventral tegmental area (VTA) is based mainly on the responses of individual putative dopamine neurons. In contrast to cortical areas, the nature of coordinated interactions between groups of VTA neurons during motivated behavior is largely unknown. These interactions can strongly affect information processing, highlighting the importance of investigating network level activity. We recorded the activity of multiple single units and local field potentials (LFP) in the VTA during a task in which rats learned to associate novel stimuli with different outcomes. We found that coordinated activity of VTA units with either putative dopamine or GABA waveforms was influenced differently by rewarding versus aversive outcomes. Specifically, after learning, stimuli paired with a rewarding outcome increased the correlation in activity levels between unit pairs whereas stimuli paired with an aversive outcome decreased the correlation. Paired single unit responses also became more redundant after learning. These response patterns flexibly tracked the reversal of contingencies, suggesting that learning is associated with changing correlations and enhanced functional connectivity between VTA neurons. Analysis of LFP recorded simultaneously with unit activity showed an increase in the power of theta oscillations when stimuli predicted reward but not an aversive outcome. With learning, a higher proportion of putative GABA units were phase locked to the theta oscillations than putative dopamine units. These patterns also adapted when task contingencies were changed. Taken together, these data demonstrate that VTA neurons organize flexibly as functional networks to support appetitive and aversive learning.

## Introduction

Dopamine neurons in the ventral tegmental area (VTA) play a central role in reward processing, conditioning, instrumental behavior, hippocampal-dependent learning, motivation, attention and working memory [1], [2], [3], [4], [5], [6], [7]. Dysregulation of dopamine neurotransmission has been implicated in many brain disorders including schizophrenia, ADHD, autism, addiction and Parkinson's disease [8], [9], [10], [11]. Despite this complex set of functions and pathologies, our understanding of information encoding by dopamine neurons in behaving animals has focused mainly on the phasic responses of single neurons to novel, rewarding, aversive or conditioned stimuli [1], [12], [13], [14], [15], [16]. While single unit responses can encode a great deal of information, interactions between and within networks of neurons can strongly affect information processing in the nervous system as well [17], [18], [19]. Although neuronal interactions and ensemble encoding have been studied extensively in cortical regions, few studies have incorporated observations of oscillatory rhythms into the function of VTA neuronal activity [20], [21].

We hypothesized that in the VTA, dynamic neural interactions support learning in an outcome specific manner. We recorded from rat VTA units during an associative learning task in which a conditioned stimulus (CS) predicted either appetitive or aversive outcomes [22]. Using this task, the impact of learning on coordinated neuronal activity in the VTA was investigated by examining the interaction between unit pairs as well as LFP oscillations. Correlations between neurons can potentially influence the total information decoded from a population [19], [23], [24], [25], [26], [27], [28]. Shared connections between neurons determine the degree to which neural activity is correlated, and changes in the correlation structure indicate that the functional connectivity between neurons has changed [29], [30], [31], [32]. Related to the correlation structure, multiple neurons can encode information synergistically, meaning information that is decoded by examining their joint activity is not decoded by examining individual responses [19]. We analyzed correlations in neural discharge between simultaneously recoded neurons and the degree of redundancy or synergy of information transmitted by pairs of neurons versus each single neuron [17], [19], [32].

In addition to examining the interactions between unit pairs, we measured LFP spectral power and phase-locking between VTA neural discharge and LFP oscillations during CS presentation. Phase-locking of spike discharge to LFP oscillations is a mechanism by which spike discharge can be organized [33] and thus may be a mechanism which influences the correlation structure and encoding scheme of VTA neural responses. Collectively, through these phenomena, neuronal activity may be organized to facilitate information processing in the VTA, and support cognitive processes that emerge from the interaction of the VTA with target regions.

## Results

**Figure 1:**
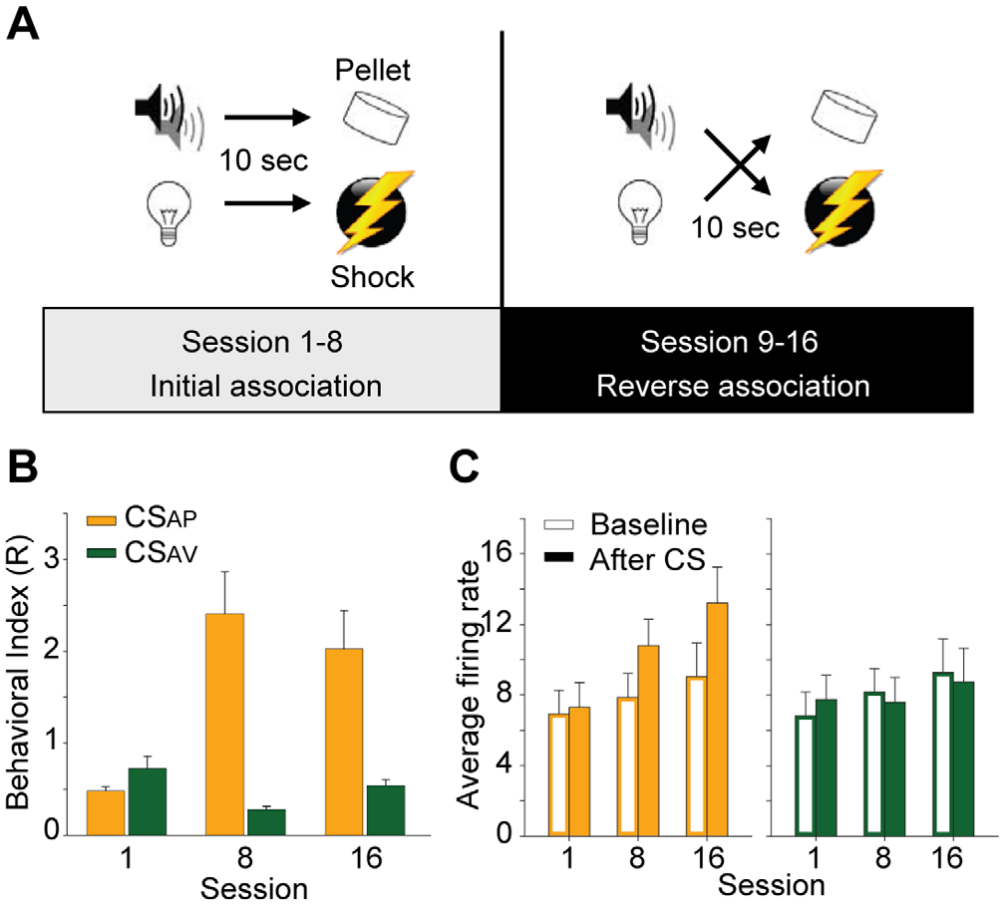
Task, behavior, and single unit activity. (**A**) Schematic representing the two conditioned associations in the initial segment of the task (left: sessions 1–8) and following the reversal of the initially conditioned associations (right: sessions 9–16). A tone or light CS was randomly presented for 10 sec and, upon termination of the stimulus, an aversive (mild electrical shock; 180 ms, 0.2 mA) or appetitive (sugar pellet 45 mg) outcome was delivered to the animal. Following 8 consecutive sessions of conditioning, these initial associations were reversed. (**B**) Behavioral performance. Data presented as mean + SEM. The behavioral index (*R*) is the ratio of nose pokes in the food trough during either CS presentation (10 sec, 30 trials) relative to nose pokes in the food trough during the baseline period (10 sec, 60 trials). (**C**) Population activity of VTA units. Data presented as mean + SEM. Empty bars represent the pre-stimulus baseline window (0.5 sec: −1 to −0.5 sec), filled bars represent the stimulus delivery window (0.5 sec: 0 to 0.5 sec). Data are plotted separately for CS_AP_ and CS_AV_ (left and right, respectively). Note that in the first conditioning session (session 1), population responses during CS_AP_ and CS_AV_ presentation did not differ from baseline levels. In the final sessions of the initial association (session 8) and reversal sessions (session 16), VTA population responses increased during CS_AP_ presentation and decreased during CS_AV_ presentation. Note that there were no statistically significant differences of firing rates during baseline period across session.

Each CS was paired with either appetitive or aversive stimuli in sessions 1–8 and these associations were reversed in sessions 9–16 (Figure 1A). Rats progressively developed greater conditioned approach to the food delivery site during the presentation of the appetitive paired CS (CS_AP_), but not the aversive paired CS (CS_AV_). In session 1, the average conditioned approach behavior ratio, *R*, (ratio of nose pokes during 10 sec CS presentation divided by nose pokes during 10 sec baseline period, *R* = 0.5 indicates equivalent approach between CS and baseline period, see methods for more details) did not diverge strongly from 0.5 for either CS (*R* = 0.483±0.044 and *R* = 0.729±0.131, CS_AP_ and CS_AV_ respectively, *P*>0.05 for all cases, *one sample t-test*). During session 8, the session before reversal, conditioned approach was higher than baseline levels during CS_AP_ presentation (Figure 1B; *R* = 2.411±0.457, *P*<0.05, *one sample t-test*) and less than baseline levels during CS_AV_ presentation (Figure 1B; *R* = 0.280±0.036; *P*<0.05, *one sample t-test*). We reversed the associations following session 8. The stimulus designated as CS_AP_ in session 8 predicted sugar pellet delivery whereas the same stimulus (tone or light) in session 16 predicted shock and is designated as CS_AV_. The behavioral responses shifted with the reversal of these associations, and by session 16, conditioned approach during the CS_AP_ was significantly higher than during the CS_AV_ (Figure 1B; *R* = 2.029±0.420 and *R* = 0.539±0.066, CS_AP_ and CS_AV_ respectively, *P*<0.05, *paired sample t-test)*. As rats developed these conditioned responses, selective response patterns emerged in VTA units. After conditioning, the CS_AP_ drove increases in population activity and the CS_AV_ decreased population activity (Figure 1C). VTA units tracked the reversal of these associations, consistent with the notion that they flexibly encode information about novel and conditioned stimuli in the environment [22]. For a more complete description of single unit and behavioral data, see [22].

### Increased correlation between VTA units after learning

**Figure 2:**
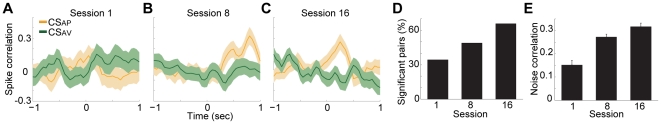
Correlated discharge between pairs of VTA units. (**A–C**) The normalized mean spike count correlation between pairs of simultaneously recorded units aligned to onset of either CS. Data are depicted as mean ± SEM (solid line = mean, shaded area = SEM). Color legend for A–C appears in upper left corner of A. (**D**) The percentage of unit pairs with statistically significant noise correlations. Data are presented as the percentage of simultaneously recorded pairs of units, which had a significant correlation in the mean subtracted spike count during each session. (**E**) Magnitude of noise correlations for significant pairs during sessions 1, 8, and 16. Data are presented as the mean noise correlation + SEM between pairs of VTA units in each session.

In fully conditioned animals (sessions 8 and 16), the CS_AP_ increased, whereas the CS_AV_ decreased correlated activity between pairs of simultaneously recorded units. This trend was not present in session 1 (Figure 2A; *P*>0.7 for CS_AP_; *P*>0.2 for CS_AV_; *t-test*), but developed with further conditioning (Figure 2B, C; *P* = 0.19 for CS_AP_ session 8; *P*<0.01 for CS_AP_ session 16; *P* = 0.06 for CS_AV_ session 8; *P*<0.01 for CS_AV_ session 16; *t-test*). Thus, after learning the initial (session 8) or reversed contingencies (session 16), VTA units discharged during the CS_AP_ in an increasingly correlated fashion, but co-varied less in response to the CS_AV_. To control for chance correlations between unit pairs, for each pair, we calculated the correlations in unit discharge between shuffled trials of spike trains. In all cases, the shuffled data sets failed to produce significant changes from baseline correlations (*P*>0.05 for all). The correlation in the residuals from the mean response to each CS (noise correlation) systematically increased with conditioning. This was evident in the percentage of pairs that were significantly correlated (Figure 2D). In session 1, 34% of pairs were significantly correlated. In conditioned animals, the percentage of unit pairs with significant correlations increased to 49% and 66% in sessions 8 and 16, respectively (*P*<0.01, *χ^2^-test*). The magnitude of the average correlation likewise increased significantly with learning (Figure 2E; *P*<0.01, *F*-test).

**Figure 3:**
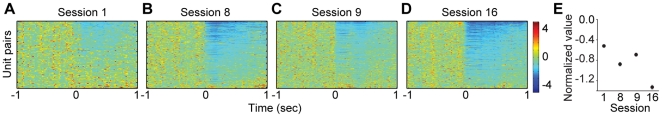
Baseline normalized information ratio (*IR*). (**A–D**) The change in *IR* aligned to CS onset. Data are depicted as mean *IR* in each time bin according to the color scale at the right. Each row represents one simultaneously recorded VTA unit pair. The data are sorted by post-stimulus normalized IR value. Each VTA unit pair is depicted in session 1 (**A**), 8 (**B**), 9 (**C**) and 16 (**D**). Note 1) the abrupt onset of the effect and 2) the uniformity of the effect, which increases dramatically from early to later sessions. **(E)** Mean information ratio of pairs of simultaneously recorded VTA units. Data are depicted as mean ± SEM and baseline normalized to 0 for sessions 1, 8, 9 and 16.

We also investigated the degree to which information was jointly encoded by pairs of VTA units relative to the amount of information encoded by each individual unit (i.e. redundancy versus synergy). We calculated a normalized information ratio (*IR*), which was simply the information about the CS encoded by the joint responses of a pair of units, divided by the quantity of information encoded by each of those units, when considered individually (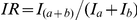; for more information on *IR*, see Methods). Overall, we found that pairs of VTA units tended to encode information redundantly, meaning that no additional information about the CS was encoded by the pair, relative to that encoded by each individual unit. Weak increases in redundancy relative to baseline were observed during the initial conditioning session (Figure 3A) but in fully conditioned animals in session 8 (Figure 3B) the decrease in *IR* became more pronounced. In the first session following reversal, *IR* still decreased relative to pre-stimulus conditions. However, this decrease was not as profound as the previous pre-reversal session (Figure 3C). In the final post-reversal session, *IR* decreased amongst nearly all unit pairs relative to pre-stimulus conditions (Figure 3D). Consistent with the effects carried at the single pair level, the mean normalized *IR* decreased throughout the learning process (Figure 3E; *IR* = −0.523±0.027, n = 131; *IR* = −0.879±0.038, n = 223; *IR* = −0.692±0.035, n = 281; *IR* = −1.329±0.042, n = 233; for sessions 1, 8, 9, and 16, respectively; *P*<0.01, *F*-test).

### CS evoked modulation of theta power

**Figure 4:**
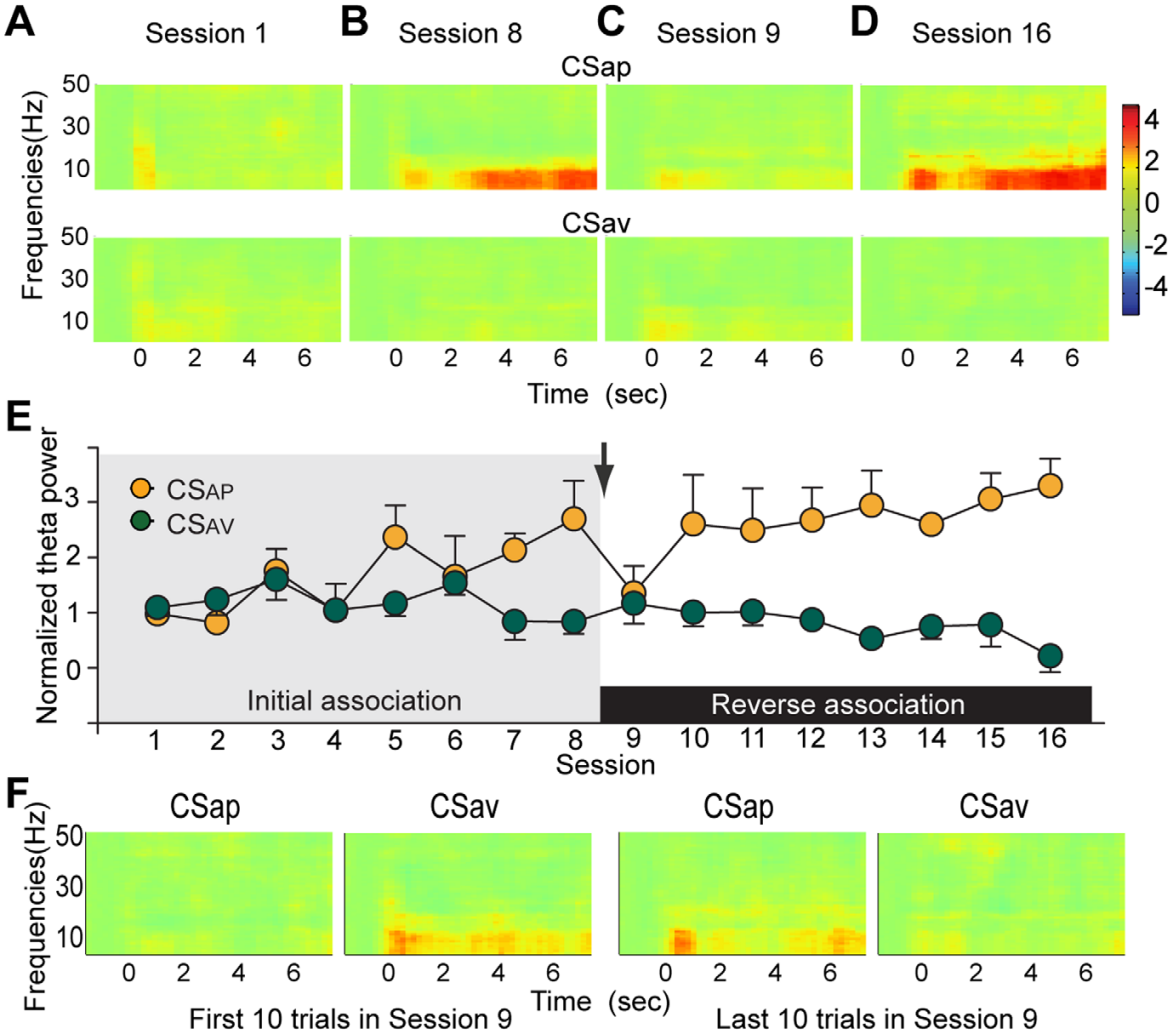
Change in LFP power pooled across subjects. (**A–D**) Spectrograms aligned to CS_AP_ and CS_AV_ and data depict group average baseline (−1.5–0 sec before CS) normalized power per frequency bin according to the color scale at the right. Frequencies between 0.7 and 50 Hz are displayed with top row representing CS_AP_ and bottom row representing CS_AV_. (**E**) Change in theta power (4–8 Hz) during a 4 second window aligned to each CS onset. Data are depicted as mean + SEM in each session. The initial associations (pre-reversal) were presented during sessions 1–8 and are depicted with the grey background. The reversed association was presented during session 9–16 and is depicted with the white background. The arrow marks the reversal and the color legend is depicted in the upper left corner. As the most prominent changes in LFP oscillations were in the theta range, we more closely examined the normalized power of theta band oscillations. (**F**) Changes in LFP power within the session 9. Data in each spectrogram are depicted as the average over the first 10 trials. Similar format and same color scale as (A–D). The CS_AP_ and CS_AV_ were reversed in session 9. Spectrograms of the first 10 trials of CS_AP_ and CS_AV_ delivery showed opposite patterns as the pre-reversal sessions, indicating that power modulations were not tracking the reversal in associations (left). In the last 10 trials, the new CS_AP_ evoked an increase in power while the new CS_AV_ modulated power in a similar fashion as pre-reversal sessions, indicating the power modulations were now tracking the reversal of associations.

Next we focused on the modulation of LFP oscillations. We first investigated modulation of broadband LFP oscillatory power in response to CS delivery. In conditioned animals, CS_AP_ but not CS_AV_ delivery, increased power in a band ranging from approximately 0–10 Hz (Figure 4A–D). We focused on our analysis on the center of this frequency range, corresponding to the theta band (4–8 Hz). This trend of CS_AP_ evoked increases in theta power was not present in sessions 1 or 9, when associations were novel (Figure 4A–D). In session 1, there was no significant difference in theta band oscillatory power between either CS (Figure 4A; 0.97±0.24 and 1.09±0.15; *P*>0.5, *t-test)*. In session 8, CS_AP_ evoked theta power was significantly greater than CS_AV_ evoked theta power (Figure 4B; 2.69±0.70 and 0.83±0.23; *P*<0.05, *t-test*). After reversal of the associations, these response patterns shifted to the new contingency. In session 9, theta band power modulation did not differ between CS_AP_ and CS_AV_ (Figure 4C; 1.35±0.50 and 1.16±0.37; *P*>0.7, *t-test*). In session 16, theta band power evoked by the CS_AP_ was significantly greater than theta power evoked by CS_AV_ (Figure 4D; 3.30±0.49 and 0.21±0.28; *P*<0.05, *t-test*). There was a significant correlation between the normalized change in theta power and session number in the pre and post-reversal sessions during CS_AP_ presentation (Figure 4E; *r* = 0.826, *P*<0.05; *r* = 0.824. *P*<0.05; initial association and reverse association, respectively). In the initial association sessions, CS_AV_ evoked theta power modulation was not significantly correlated with session number (Figure 4E; *r* = −0.394, *P*>0.3). Post-reversal CS_AV_ evoked changes in theta power were weak, though were significantly correlated with session in the post-reversal block (Figure 4E; *r* = −0.857, *P*<0.01). This effect was driven by the fact that the initially weak modulation of theta power gradually diminished as learning progressed. We looked more closely at the change of LFP power across trials in session 9, the first session following the reversal. In the first 10 trials of CS_AP_ and CS_AV_ presentation, we found that modulations of LFP power had not yet adapted to the reversed contingencies (Figure 4F, Left). In the last 10 trials of CS_AP_ and CS_AV_ presentation, we observed that LFP power modulations tracked the newly established contingencies (Figure 4F, Right).

### Phase locking between theta band LFP and spike discharge

**Figure 5:**
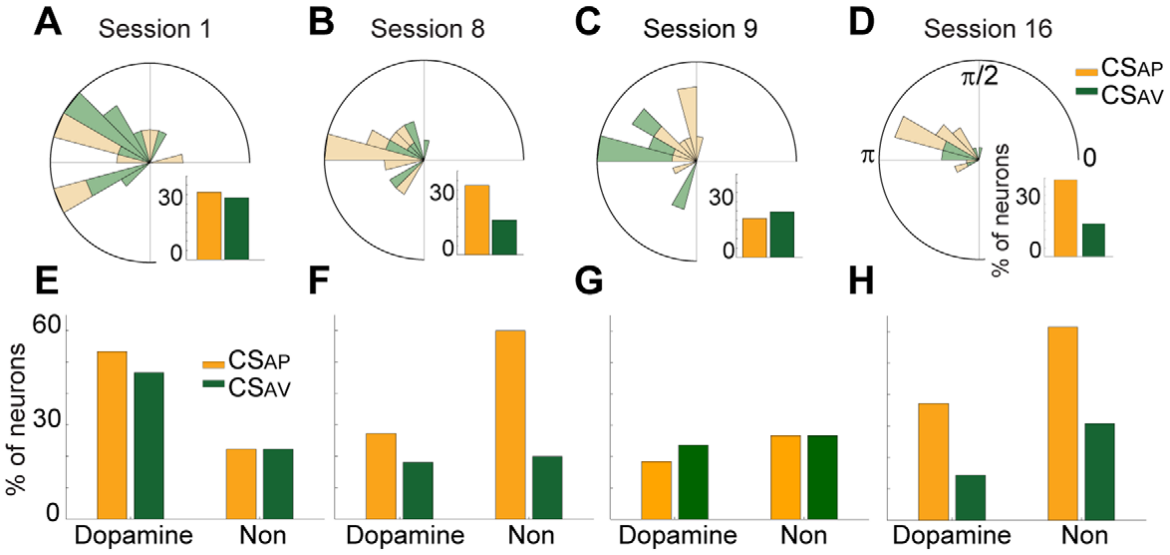
Phase locking of spike discharge to the theta rhythm. (**A–D**) Phase locking between neural discharge and LFP oscillations. Circular plots depict the number of units that were significantly phase-locked and the mean preferred angle of each unit in units of radians (bin size = π/12). Color legend is depicted at left of top row. Inset bar graphs depict the percentage of the units phased locked to the LFP theta rhythm during each CS. In session 1 (**A**), outer edge of circle inset = 3 units. In session 8 (**B**), outer edge of circle inset = 5 units. In session 9 (**C**), outer edge of circle inset = 5 units. In session 16 (**D**), outer edge of circle inset = 8 units. (**E–H**) Theta phase locking of putative dopamine and non-dopamine VTA units. The percentage of units phase locked to the theta oscillation during CS_AP_ and CS_AV_ presentation. Units were classified as putative dopaminergic or non-dopaminergic units. Data are presented with same conventions as insets from A–D.

One potential mechanism that organizes neural discharge is phase-locking between spikes and LFP oscillations [33]. During initial presentation of either the CS_AP_ or CS_AV_, a substantial proportion of units were phase-locked to the theta rhythm. In session 1 (Figure 5A), there was no difference in the proportion of units phase locked to theta band oscillations between the CS_AP_ and CS_AV_ (*P*>0.7, *χ^2^-test*; 37% (12/33), 33% (11/33) for CS_AP_ and CS_AV_ respectively). However, in conditioned animals, the relationship was modulated such that the CS_AP_ recruited a larger proportion of significantly phase-locked units than the CS_AV_ (Figure 5B). In session 8, 38% (18/48) of units phase locked during CS_AP_, while only 19% (9/48) of units phase locked during CS_AV_ (*P*<0.05, *χ^2^-test*). These patterns of phase locking flexibly tracked the reversal of associations so that in the initial session after reversal (session 9), there were equivalent proportions of units phase-locked to the theta rhythm during each CS delivery (Figure 5C; 21% (11/53) phase locked during CS_AP_; 25% (13/53) phase locked during CS_AV_; *P*>0.05, *χ^2^-test*). Once this new association was well-learned (session 16), a greater proportion of units were phase-locked to the theta oscillation during delivery of the CS_AP_ than the CS_AV_ (Figure 5D; 44% (21/48) and 19% (9/48) phase locked to the CS_AP_ and CS_AV_, respectively in session 16; *P*<0.01, *χ^2^-test*). There was no difference in the average preferred phase angle between sessions or CS (0.85π±0.24, 0.83π±0.22 in session 1; 0.94π±0.13, 0.81π±0.24 in session 8; 0.87π±0.09, 0.86π±0.20 in session 16 for CS_AP_ and CS_AV_, respectively; *P*>0.4 for both session and CS, Watson-Williams test).

For the phase-locking analysis, we classified the phase-locked units as putative dopaminergic and non-dopaminergic units (Figure 5E–H), as previously described [22]. In session 1, more putative dopamine units were phase locked during either CS than were non-dopaminergic units (Figure 5E; 53%, (8/15); 47%, (7/15); putative dopamine CS_AP_ and CS_AV_, respectively; 22%, (4/18); 22%, (4/18); non-dopamine CS_AP_ and CS_AV_, respectively). However, in fully conditioned animals (sessions 8 and 16), the majority of units that were phase-locked to the theta rhythm were non-dopaminergic and this relationship mostly driven by phase-locking during CS_AP_ delivery. In session 8 (Figure 5F), more putative non-dopamine (60%, 9/15) than dopamine units (27%, 9/33) were phase-locked during CS_AP_ delivery. Similarly, in session 16 (Figure 5H), 62% (8/13) of putative non-dopamine and 37% (13/35) of dopamine units were phase locked during the CS_AP_.

## Discussion

These data demonstrate that VTA neurons flexibly organize as functional networks that differentiate the appetitive and aversive nature of expected outcomes. We observed that correlated discharge between VTA unit pairs increased during presentation of CS_AP_ relative to CS_AV_. This pattern emerged after conditioning and tracked the reversal of associations. A strong noise correlation between VTA unit pairs developed as conditioning progressed. The joint information content between VTA unit pairs became increasingly redundant in conditioned animals. We also observed that CS_AP_, but not CS_AV_, was associated with strong, learning dependent increases in theta power that persisted throughout the delivery of the CS. During the first conditioning session, equivalent proportions of units were phase-locked to the theta oscillation during presentation of either CS. With further conditioning, the CS_AP_ recruited a greater number of putative GABA VTA units into theta phase-locking than the CS_AV_. This trend also tracked the reversal of associations.

Recordings from individual VTA and substantia nigra neurons have previously established that discharge rates are modulated in response to stimuli predictive of appetitive or aversive outcomes [1], [15], [16], [34], [35], [36] and these responses develop as a function of learning [12], [14], [22]. We found that, in conjunction with this change in discharge rate, VTA neuronal discharge is increasingly correlated in response to stimuli predictive of an appetitive outcome, indicating that reward predictive stimuli engage mechanisms that increase the covariance of VTA neurons. It has been suggested that dopamine neuron containing midbrain regions may specialize in encoding positive outcomes or stimuli predictive of positive outcomes [36], [37]. The increased correlation in discharge between VTA neurons that we observed, may impact the ability of appetitive CS information to be decoded from the population [23], [26], [28]. Thus, assuming that neural representations of reward prediction errors or other learning related signals are decoded from VTA population activity, this correlation structure could strongly shape the rate of learning related to outcome expectation.

The residual from the mean (noise) spike count also became more correlated between pairs of VTA neurons across learning. Correlated discharge between neurons is generally reflective of shared inputs [29], [30], [31], [32], suggesting that shared functional connectivity of VTA neurons increase with learning. While our analysis does not allow us to infer if new neurons in the population are coming online or if the same neurons are changing connectivity patterns, the average degree of functional connectivity increased within the population as learning occurred. These data suggest that modification of VTA network connections may be an important component of conditioning: as environmental stimuli acquire a predictive value, in order for VTA neurons to acquire new response patterns, new patterns of connectivity must be established.

The responses of VTA neurons became highly redundant after conditioning. It has been widely observed that the responses of dopamine neurons are homogenous [1], [38]. Additionally, our previous work suggests that putative GABA and dopamine VTA neurons have similar CS evoked responses, indicating this homogeneity is not restricted by neurotransmitter content [22]. The present data suggests that, not only do most neurons in the VTA have similar stimulus mean evoked responses, but when simultaneously recorded neural activity is examined, redundant encoding emerges. To coordinate the discharge of a large number of neurons into a redundant signal requires extensive resource allocation [39]. While the resulting signal is more robust and resistant to degradation, the total information carried by the signal may be greatly decreased [19], [23], [26], [32], [40]. The costs of such an arrangement may emphasize the importance of this encoding scheme. Learning theories suggest that similar reinforcement learning signals are broadcast to multiple target regions [38], [41]. Since VTA projections rarely collateralize between target regions [42], redundant signaling would ensure that similar signals reach each region. The redundant VTA signal may also simplify the organizational demands of the region by reducing the need to create post-synaptic convergence between cells with disparate information content. This coding scheme may be optimal for promoting rapid learning or behavioral flexibility as fewer neurons need to acquire a response pattern or gain connectivity before effective signaling can be established.

The power of theta oscillations in the VTA selectively increased during CS predictive of appetitive, and not aversive outcomes. In human studies, increased power of theta oscillations has been observed to occur across the duration of single trials, possibly “gating” changes in context [43]. Similarly, in the current study, increased power theta oscillations persisted through the delivery of the CS_AP_ though most VTA neurons responded to these stimuli with short-duration, short-latency modulation of discharge rate. In this case, VTA theta rhythms may gate periods of time during which there is an expectation of appetitive or rewarding outcomes. Interestingly, increased theta power or phase synchrony has been reported in several prefrontal cortex subregions during conditioned stimulus and reward delivery or expectation [44], [45]. Moreover, coherent theta rhythms in the hippocampus and striatum are implicated in the acquisition and performance of behavior as well as synaptic modification [46], [47], [48], [49]. In addition to a specific role in reward processing, theta rhythms have been suggested to support long-range synchronization between brain regions and inter-region communication [50], [51]. Thus, the modulation of theta power observed here may serve to synchronize the activity of networks encoding information during periods of reward anticipation or availability, and may be reflective of inter-region processing necessary for learning.

Only a few studies have investigated oscillatory phenomena in the VTA. In anesthetized rats, VTA dopamine neurons demonstrated delta band oscillations in discharge rate that are dependent upon input from prefrontal cortex, and coherent with LFP oscillations in the PFC [52], [53]. Similarly, cortical up-states and VTA LFP oscillations are coherent in the delta frequency range [20]. The oscillations observed in these studies occur in lower frequency ranges than that which we observed in the current study. One potential explanation for this difference is that anesthesia could alter the patterns of afferent drive upon these networks, and reduce the dominant spectral components. In behaving animals, prefrontal cortex and VTA coherently oscillate in a frequency range centered at 4 Hz, and extending from 2–5 Hz during periods of working memory maintenance [21]. Oscillations at 4 Hz and above were included in our definition of theta, though our definition extended to 8 Hz. It is unclear what underlies the differences in frequency ranges between our study and the aforementioned study. One possibility is that different cognitive functions, such as working memory and Pavlovian conditioning, engage oscillations in slightly different frequency ranges to facilitate communication between VTA and prefrontal cortex. Differences in the frequency range of the dominant spectral components aside, these data are consistent with our suggestion that the VTA potentially uses slow (less than 10 Hz) rhythmic LFP oscillations to integrate information processing with the prefrontal cortex.

Phase locking of spike discharge to theta oscillations may serve to precisely control the timing of VTA phasic signaling. Dopamine neurons signal errors in the timing of when rewards were predicted to occur [1], [54]. Detection of stimuli, and comparison with when these signals should appear, requires a time keeping mechanism [41], [55] and the rhythmic nature of LFP oscillations could provide an electrophysiological time signal in the range of several hundred milliseconds. Thus, VTA theta rhythms and phase locking between these ongoing oscillations and spike discharge, may control spike timing and facilitate precise temporal communication. Theta phase locking may also provide a mechanism that produces correlated discharge among VTA neurons. Theta band phase-locking could regulate the timing of spike discharge and thus produce more correlated patterns of discharge between neurons. This may ultimately optimize VTA signaling.

Our data show that while the average discharge patterns of putative dopamine and GABA neurons are similar during conditioning, there could be a functional distinction between these groups on the basis of phase-locking. The majority of neurons that phase-locked to the theta oscillation in session 1 were putative dopamine units, whereas in sessions 8 and 16, the majority of phase-locked neurons were putative GABA units. Dopamine neurons in the VTA receive robust sensory input from lower brain structures [54], [56], potentially underlying phase-locking during the presentation of novel and unconditioned stimuli. While little is known about the function GABA neurons of the VTA, these neurons (and not dopamine neurons) receive synaptic input from the nucleus accumbens [57], a region that is critical to reward processing [3]. These patterns of connectivity and the current data suggest that phase-locking of VTA GABA neurons supports reward related learning.

The present results have implications for dopamine related disorders. The redundant nature of VTA signaling could reduce the impact of cell death and provides a potential mechanism for why destruction of over 50 percent of midbrain dopamine neurons is necessary for symptom expression in Parkinson's disease [58]. The redundant and coordinated activity of the remaining dopamine neurons may provide a sufficient signal to overcome the loss of other dopamine neurons. In contrast, in cognitive disorders such as schizophrenia, dopamine related dysfunctions occur in the absence of obvious pathology in dopamine neurons or receptors [9]. Our data suggest that precise control of VTA neural interactions is critical to VTA function. Since disrupted oscillatory activity has been reported extensively in schizophrenia [59], dopamine related dysfunction may arise from disrupted rhythmic oscillations and integration of VTA signals rather than gross morphological disruptions of individual neurons.

In conclusion, we examined network and pair-wise interactions that characterize information processing in the VTA. With the exception of a few studies, this area has received far less attention than the manner in which individual VTA neurons process information. We suggest that these interactions within the VTA may shape how information is encoded by VTA neurons, and how information is decoded from the target projections of VTA neurons. Ultimately, the rhythmic brain oscillations that are present in the VTA may serve to organize spike discharge patterns and orchestrate neuronal interactions within the VTA and between the VTA and other brain regions. The redundant patterns of neural activity observed in the VTA may produce a unified signal that allows multiple target regions to receive similar information about environmental stimuli, from which a diverse array of cognitive functions can be guided.

## Materials and Methods

### Subjects, surgery, behavioral task, data acquisition

Experimental procedures for animal behavior, surgery, physiological recording, isolation and classification of unit signals, and histology were described previously [22]. Similarly, behavior and CS evoked single unit discharge from this data set have been previously described [22]. In the current paper, we analyzed LFP data obtained simultaneously with single unit data, and analyzed the joint responses of pairs of VTA neurons. Briefly, seven adult male Sprague-Dawley rats (300–360 g) were chronically implanted with bilateral 8 channel microelectrode arrays (NB Laboratories, Denison, TX, USA) in the VTA (target coordinates for the center of array, relative to Bregma: AP −5.3 mm, ML 0.5–1.1 mm , DV 7.7–8.3 mm [60]. After recovery from surgery, rats were habituated to the experimental chambers (Coulbourn Instruments, Allentown, PA, USA). After 2 days of habituation, animals began the first session of a conditioning task. During sessions 1–8, two different CS were presented, either a 10 sec duration tone or flashing light. In a counter-balanced fashion, each was paired with either an appetitive stimulus (a sugar pellet US_AP_; Bioserv dustless precision pellets 45 mg, Frenchtown, NJ, USA) or an aversive stimulus (mild electric shock US_AV_; 180 ms, 0.2 mA) through a stainless steel grid floor. These outcomes immediately followed the termination of either CS. Rats underwent 60 stimulus pairings (30 trials of each contingency) delivered pseudo-randomly with a 20 sec inter-trial interval (Figure 1A). After eight sessions, the initial associations were reversed in sessions 9–16. The experimental procedures and behavioral recordings were performed with Graphic State software (Coulbourn Instruments, Allentown, PA, USA). All procedures were in accordance with the University of Pittsburgh's Institutional Animal Care and Use Committee.

Recordings were made during daily behavioral sessions. Signals passed through a unity-gain JFET headstage amplifier before being analog filtered (single units: 0.3–8 KHz band pass, LFP: 0.7–170 Hz) and amplified 1000× (Plexon, Dallas, TX, USA). The activity from multiple simultaneously recorded single-units was digitally high-pass filtered at 300 Hz and LFP was low-pas filtered at 125 Hz. Data was stored to disk for offline analysis. Single units were isolated in Offline Sorter (Plexon, Dallas, TX, USA).

### Behavioral Analysis

We used the ratio of nose pokes into the food trough during CS presentation and baseline as an index of conditioned behavior, termed *R*. For each recording session, the number of food trough nose pokes was tabulated during each CS_AP_ and CS_AV_ presentation interval: CS_AP_ (10 sec) ×30 trials, CS_AV_ (10 sec) ×30 trials. This measure was normalized by dividing the number of nose pokes during each CS by the number of nose pokes during the second half of each 20 sec intertrial interval (10 sec) ×60 trials (if no bias in the approach occurred relative to intertrial interval, *R* = 0.5). Instead of using only the 30 intertrial intervals preceding each specific CS, we pooled the nose pokes from all 60 intertrial intervals for this measure (there was no signiicant difference in the number of nose pokes preceding the CS_AP_ and CS_AV_, 17.66±1.39 and 18.44±1.46, *t-test*, *P*>0.05).

### Neuronal correlation and information ratio

We simultaneously recorded 131, 223, 281 and 233 neuron pairs (pooled across rats) in sessions 1, 8, 9 and 16, respectively. The correlation between unit discharge, and mutual information between unit discharge and stimulus were analyzed. For these analyses, we did not group unit pairs based on putative neurotransmitter content in order to preserve sufficient sample sizes for reliable analysis. Thus, our data on pair-wise interactions between VTA units are summaries of all simultaneously recorded pairs of VTA units, irrespective of putative neurotransmitter content. All spike train analysis utilized custom scripts executed in the Matlab environment (MathWorks, Natick, MA). We correlated the trial-by-trial fluctuations in discharge rate between simultaneously recorded pairs of neurons. A Pearson's correlation of spike counts for each pair of units was calculated in the time period extending ±1 sec around either CS. Correlations were computed in a 200 ms sliding window, advanced in 50 ms steps. In the text, this analysis is referred to as “spike count correlation”. To control for chance correlations, we randomly shuffled all trials of spike count data from each unit, in each unit pair. This removed the physiologically meaningful correlation in spike discharge between unit pairs, and any remaining correlation was considered an artifact of the discharge patterns of each unit. Comparison of the actual correlation and shuffled correlation values revealed that artifactual correlations did not bias the data strongly.

Noise was defined as each unit's trial-to-trial residual from the average CS evoked spike count. We calculated the trial-by-trial correlation in “noise” (mean-subtracted) spike counts between each pair of simultaneously recorded units. For this analysis, we measured the correlation in the time period ±1 sec surrounding each CS. Non-overlapping 500 ms windows were utilized. In the text, this analysis is referred to as “noise correlation”. We use the terminology noise for consistency with previous literature, and acknowledge that our quantification of noise makes no assumptions or indications of the cause of the deviation from the mean [19], [32].

We also calculated the mutual information between neural discharge and stimuli. Briefly, spike counts in the time period ±1 sec around each stimulus were used to calculate mutual information in a 200 ms moving window, advanced in 20 ms steps, using the following equation:
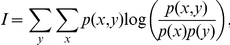
Where x is the stimulus, y is the spike count, and *I* is the mutual information between stimulus and spike count.
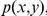
is the joint probability of stimulus x and spike count y, and
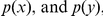
are the marginal probabilities of x and y, respectively.

The information transmission of pair of units that were recorded simultaneously also was calculated using the same formula as above, except that the spike count y represented the joint spike count of each unit. Similar to previous reports [17], [32], [61], the synergy or redundancy of information transmission was assessed by calculating the information ratio (*IR*):
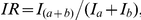
where *I_a_* and *I_b_* represent mutual information about each stimulus separately transmitted by units a and b, *I*_(*a+b*)_ indicates the information jointly transmitted by two spike trains. All calculations of *I are derived from the formula listed above*. Cue evoked changes in *IR* were Z-score normalized against a 1 sec pre-stimulus baseline using the mean and standard deviation *IR* values. If more information was encoded by the joint activity of the pair, as compared to the sum of information encoded by each unit separately, the pair encoded information synergistically. If the same amount of information was encoded by the joint activity of the pair, as was encoded by each unit separately, the pair encoded information redundantly.

### Local field potential

All LFP analysis utilized custom scripts based on routines from the Chronux toolbox (chronux.org) in the Matlab environment (MathWorks, Natick, MA), using standard techniques [62]. LFP data surrounding each CS (−2 sec to +8 sec peri-event window) were analyzed. Possible DC offsets, linear trends, 60 Hz line noise, and data segments containing obvious movement artifacts were removed. Spectral power for each trial during a 10 second period was calculated via multitaper Fourier transform in a 1 sec moving window, advanced in 200 ms steps. A standard multi-taper approach was utilized with the 13 leading tapers, allowing for a frequency resolution of 1 Hz. Each frequency bin in the power spectrum was Z-score normalized against the baseline period. We display changes in LFP power across the entire frequency range from 0–50 Hz for clarity. Our analysis focuses exclusively on the theta band of the frequency spectrum (4–8 Hz).

### Phase locking

We analyzed spike-LFP phase locking between both putative dopamine and non-dopamine units in VTA. Our analyses were comprised of 33 units (15 dopamine, 18 for non-dopamine) in session 1, 48 units (33 dopamine, 15 non-dopamine) in session 8, 53 units (38 dopamine, 15 non- dopamine) in session 9 and 48 units (35 dopamine, 13 non-dopamine) in session 16 (all units pooled across animals). To determine phase locking of neural discharge to LFP, LFP data was wavelet transformed (Morlet wavelet) and the phase angle of the LFP oscillation at the time of spike discharge was calculated using custom Matlab routines. Data are reported in radians between 0 and 2π aligned to the oscillation peak. A neuron was considered phase-locked if the distribution of spike phase angles was non-uniform (Rayleigh's test, p<0.05). The number of neurons that were phase-locked were plotted on a unit circle with (bin size = π/12). The proportion of units phase-locked in different CS was compared in sessions 1, 8, 9 and 16.
